# Cancer Treatment: New Drugs and Strategies

**DOI:** 10.3390/ijms25189976

**Published:** 2024-09-16

**Authors:** Nuno Vale

**Affiliations:** 1 PerMed Research Group, Center for Health Technology and Services Research (CINTESIS), Rua Doutor Plácido da Costa, s/n, 4200-450 Porto, Portugal; nunovale@med.up.pt; Tel.: +351-220-426-537; 2 CINTESIS@RISE, Faculty of Medicine, University of Porto, Alameda Professor Hernâni Monteiro, 4200-319 Porto, Portugal; 3 Department of Community Medicine, Information and Health Decision Sciences (MEDCIDS), Faculty of Medicine, University of Porto, Rua Doutor Plácido da Costa, s/n, 4200-450 Porto, Portugal

Cancer is a significant global health problem with complex treatment challenges. Recent innovative approaches such as nanomedicines, combined therapy, drug repurposing, genomics, and personalized therapy have strategically targeted the hallmarks of cancer, disrupting key processes that drive tumor growth and spread [1,2]. These strategies include in silico studies, advanced data analysis, and translation research aimed at precisely inhibiting cancer progression with minimal harm to healthy cells and potentially enhancing the immune system’s response to tumors [3,4]. This Special Issue delves into these advancements, presenting nine original research papers of immense potential and three reviews on very current themes. Each paper explores innovative methods that address specific cancer mechanisms, demonstrating the evolving landscape of oncological research.

Dr. Sabrina Reis’ study on prostate cancer (PCa) exemplifies such an innovative inflammation control strategy. Utilizing CRISPR-Cas9 genome editing, her research targets genes MMP9 and miR-21, both associated with PCa progression. By editing these genes in PCa cell lines, the study observed reduced cell proliferation, increased apoptosis, and decreased invasion, highlighting the potential of CRISPR-Cas9 in developing effective cancer therapies. This combined insight underscores the importance of novel therapeutic strategies in advancing cancer treatment. An-Jing Ren’s study explores the role of FKBP10, a protein linked to cancer development, in colorectal cancer (CRC). Analyzing data from online databases and a CRC cohort, the study identified three subcellular expression patterns of FKBP10 in tumor tissues: ‘FKBP10-C’ (concentrated), ‘FKBP10-T’ (transitional), and ‘FKBP10-D’ (dispersive). The FKBP10-D pattern, found only in tumors, was associated with poor disease-free survival. High levels of FKBP10-C predicted an unfavorable prognosis for CRC recurrence. These findings suggest that FKBP10’s expression patterns and levels are crucial prognostic indicators, making it a potential target for CRC diagnosis and treatment. Jelena Ostojic investigated the potential of Slug (Snail2) and Snail (Snail1), zinc finger transcriptional factors involved in tumor progression as potential staging and prognostic markers in renal cell carcinoma (RCC), a highly lethal urological cancer currently lacking validated biomarkers for screening and follow-up. Analyzing 166 RCC samples, this study found that Slug expression significantly impacts survival, with Slug identified as an independent prognostic factor. Snail expression was linked to higher disease stages and metastasis, particularly nuclear Snail. These findings suggest that Slug and Snail could serve as valuable immunohistochemical markers and potential therapeutic targets for RCC.

As part of strategies focused on immunomodulation, Wafik S. El-Deiry’s study examines the GSK-3 inhibitor elraglusib (9-ING-41) for its immunomodulatory effects in CRC. Elraglusib was found to enhance the immune-cell-mediated killing of CRC cells by sensitizing them to immune cytotoxicity and boosting immune effector functions. It reduced the expression of survival proteins and immunosuppressive molecules in CRC cells while increasing proapoptotic genes and immune cell IFN-γ secretion. In a murine model, elraglusib combined with anti-PD-L1 therapy improved survival and increased tumor-infiltrating T cells. Clinically, elraglusib-treated patients showed reduced immunosuppressive markers and elevated immune activation markers, suggesting its potential in combination therapies for CRC and other tumors. Similarly, the study conducted by Sayed K. Goda focused on using bacterial superantigens (SAgs) for immunotherapy due to their potent T-cell activation capabilities. Superantigens activate a large percentage of T cells, unlike conventional antigens. However, their high lethality poses a challenge. The study aims to create safer superantigen-based peptides for cancer treatment with minimal toxicity. Researchers designed and synthesized SPEA-based peptides, identifying regions responsible for T-cell activation and tumor killing. Tumor-targeted superantigen-based peptides were also developed, demonstrating specific cancer cell binding and killing. These findings suggest that combining these peptides with other immunotherapies could lead to effective and safer cancer treatments. Subburaj Ilangumaran’s study focuses on overcoming tumor immune evasion by restoring MHC class-I (MHC-I) expression via NLRC5, a key regulator of antigen-processing genes. In poorly immunogenic B16 melanoma cells, reintroducing NLRC5 enhances MHC-I expression, promoting antitumor immunity and suggesting NLRC5 as a potential immunotherapy target. To address NLRC5’s large size as a constraint for clinical use, the study explores a smaller NLRC5-CIITA fusion protein, NLRC5-superactivator (NLRC5-SA), which retains MHC-I induction capability. The stable expression of NLRC5-SA in mouse and human cancer cells effectively upregulates MHC-I expression and controls tumor growth comparable to full-length NLRC5 (NLRC5-FL). The mass spectrometry analysis of MHC-I-associated peptides indicates that NLRC5-SA broadens the peptide repertoire, potentially enhancing tumor immunogenicity beyond NLRC5-FL. This suggests NLRC5-SA as a promising candidate for advancing translational immunotherapy applications.

Robin Farias-Eisner investigates HM-10/10, a 20-amino acid peptide, as a prospective treatment for ovarian and colon adenocarcinomas. The peptide demonstrates robust stability in human plasma and simulated gastric conditions, positioning it as a potential oral pharmaceutical. Challenges in intestinal environments prompt ongoing efforts to enhance its stability and bioavailability. This research underscores HM-10/10’s potential to address critical women’s health challenges associated with these cancers.

Rodrigo A. López-Muñoz explores the analysis of drug combinations in anticancer research, focusing on the comparison between Combenefit and SynergyFinder. This study examines combinations of non-steroidal analgesics (celecoxib and indomethacin) with antitumor drugs (carboplatin, gemcitabine, and vinorelbine) on canine mammary tumor cell lines. Mathematical models (HSA, Loewe, Bliss) are applied to viability data to assess synergy. Combenefit shows stronger synergy signals, while SynergyFinder excels in concentration–response fitting. Differences in software algorithms influence synergy outcomes, often altering combination classification from synergistic to antagonistic based on curve fitting. Normalization using simulated data reveals that Combenefit enhances the distinction between synergy types more than SynergyFinder. The study emphasizes the need for comprehensive data analysis and the use of multiple models in drug combination studies to accurately interpret synergy.

Magdalena Stobiecka presents a novel fluorometric assay using an aptamer beacon probe (ABP) for detecting adenosine triphosphate (ATP) as an energy biomarker in cancer screening. The ABP demonstrated sensitivity and rapidity in detecting ATP levels, crucial for assessing malignancy risk. Testing included ATP solutions and other nucleotides, followed by application in SW480 cancer cells to monitor ATP production. Temperature stability studies showed optimal performance at 40 °C, enhancing ABP selectivity for ATP. Inhibiting glycolysis in SW480 cells with 2-deoxyglucose reduced ATP production by 31.7%, suggesting potential for ATP modulation in cancer treatment strategies.

In the category of review articles, this Special Issue addressed three perspectives. Carlotta Sacerdote’s review focuses on circulating tumor cells and tumor-derived materials like circulating tumor DNA and circulating miRNAs (cfmiRNAs) in cancer research. The review centers on cfmiRNA studies in breast cancer, aiming to identify diagnostic and prognostic biomarkers through the systematic analysis of 16 publications. Despite revealing differential expressions of cfmiRNAs such as MIR16 and MIR191 between breast cancer cases and healthy controls, the studies are limited by bias and lack standardized protocols. The findings underscore the need for improved study designs and larger sample sizes to validate cfmiRNAs’ potential as clinical diagnostic tools in breast cancer. Our review explores the intricate role of human endogenous retroviruses (HERVs) [5], particularly HERV-K (HML-2), in cancer development and progression. It discusses recent research advancements and potential treatment strategies, examining HERVs’ historical context and their impact on key biological processes such as embryonic development, immune response, and disease progression. The review encompasses computational modeling for assessing drug-target binding, systems biology modeling to simulate HERV-K viral cargo dynamics, and the use of antiviral drugs against HERV-associated diseases. These insights enhance our understanding of HERV-mediated disease mechanisms and offer perspectives on future therapeutic approaches, highlighting HERV-K’s potential as both a biomarker and therapeutic target. Jelena Milovanovic’s review focuses on the inflammatory mechanisms driving the formation and progression of oral squamous cell carcinoma (OSCC), emphasizing the role of the proinflammatory cytokine IL-17. IL-17 is implicated in tumor development, growth, and metastasis, as evidenced in both experimental models and OSCC patients where its presence correlates with increased cancer cell proliferation and invasiveness. The review explores IL-17’s influence on OSCC pathogenesis, including its role in stimulating the production of proinflammatory mediators that activate myeloid cells with suppressive and proangiogenic functions, as well as its direct promotion of cancer cell and stem cell proliferation. Additionally, the potential for IL-17 blockade as a therapeutic strategy in OSCC is discussed, highlighting its promise for future clinical applications.
